# 2-Fluoro-*N*-(4-methoxy­phen­yl)benzamide

**DOI:** 10.1107/S1600536809008927

**Published:** 2009-03-19

**Authors:** Aamer Saeed, Rasheed Ahmad Khera, Madiah Arfan, Jim Simpson, Roderick G. Stanley

**Affiliations:** a Department of Chemistry, Quaid-i-Azam University, Islamabad 45320, Pakistan; b Department of Chemistry, University of Otago, PO Box 56, Dunedin, New Zealand

## Abstract

In the title compound, C_14_H_12_FNO_2_, the fluoro­benzene and methoxy­benzene rings are inclined at 27.06 (7) and 23.86 (7)°, respectively, to the amide portion of the mol­ecule and at 3.46 (9)° to one another. The meth­oxy substituent lies close to the methoxy­benzene ring plane, with a maximum deviation of 0.152 (3) Å for the methyl C atom. In the crystal structure, inter­molecular N—H⋯O hydrogen bonds link mol­ecules into rows along *a*. Weak C—H⋯O and C—H⋯F inter­actions further stabilize the packing, forming corrugated sheets in the *bc* plane.

## Related literature

For the biological activity of benzamides and related compounds and their use in organic synthesis, see: Vega-Noverola *et al.* (1989 ▶); Yoo *et al.* (2005 ▶); Saeed *et al.* (2008 ▶). For related structures, see: Saeed *et al.* (2008 ▶); Chopra & Guru Row (2008 ▶); Donnelly *et al.* (2008 ▶); Cockroft *et al.* (2007 ▶); Spitaleri *et al.* (2004 ▶).
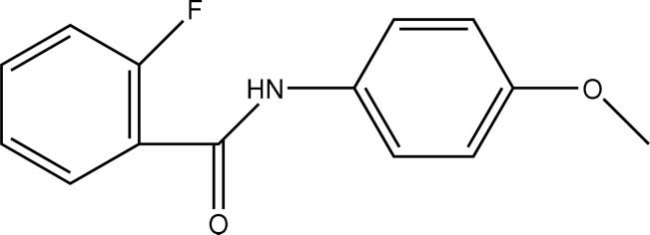


## Experimental

### 

#### Crystal data

C_14_H_12_FNO_2_
*M* *_r_* = 245.25
Orthorhombic, 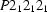

*a* = 5.2901 (2) Å
*b* = 6.6435 (3) Å
*c* = 31.7823 (11) Å
*V* = 1116.98 (8) Å^3^
*Z* = 4
Mo *K*α radiation
μ = 0.11 mm^−1^
*T* = 89 K
0.30 × 0.23 × 0.04 mm

#### Data collection

Bruker APEXII CCD area-detector diffractometer
Absorption correction: multi-scan (*SADABS*; Bruker, 2006 ▶) *T*_min_ = 0.853, *T*_max_ = 0.996
19727 measured reflections
2185 independent reflections
1980 reflections with *I* > 2σ(*I*)
*R*_int_ = 0.049

#### Refinement

*R*[*F* ^2^ > 2σ(*F* ^2^)] = 0.035
*wR*(*F* ^2^) = 0.096
*S* = 1.06
2185 reflections
168 parameters
H atoms treated by a mixture of independent and constrained refinement
Δρ_max_ = 0.37 e Å^−3^
Δρ_min_ = −0.24 e Å^−3^

### 

Data collection: *APEX2* (Bruker 2006 ▶); cell refinement: *APEX2* and *SAINT* (Bruker 2006 ▶); data reduction: *SAINT*; program(s) used to solve structure: *SHELXS97* (Sheldrick, 2008 ▶) and *TITAN2000* (Hunter & Simpson, 1999 ▶); program(s) used to refine structure: *SHELXL97* (Sheldrick, 2008 ▶) and *TITAN2000*; molecular graphics: *SHELXTL* (Sheldrick, 2008 ▶) and *Mercury* (Macrae *et al.*, 2006 ▶); software used to prepare material for publication: *SHELXL97*, *enCIFer* (Allen *et al.*, 2004 ▶), *PLATON* (Spek, 2009 ▶) and *publCIF* (Westrip, 2009 ▶).

## Supplementary Material

Crystal structure: contains datablocks global, I. DOI: 10.1107/S1600536809008927/si2159sup1.cif

Structure factors: contains datablocks I. DOI: 10.1107/S1600536809008927/si2159Isup2.hkl

Additional supplementary materials:  crystallographic information; 3D view; checkCIF report

## Figures and Tables

**Table 1:** Hydrogen-bond geometry (Å, °)

*D*—H⋯*A*	*D*—H	H⋯*A*	*D*⋯*A*	*D*—H⋯*A*
N1—H1*N*⋯O1^i^	0.84 (2)	2.34 (2)	3.1366 (17)	158.5 (17)
C4—H4⋯F1^ii^	0.95	2.44	3.2472 (17)	143
C14—H14*B*⋯O2^iii^	0.98	2.61	3.3753 (18)	135

Symmetry codes: (i) 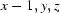; (ii) 
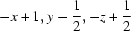
; (iii) 
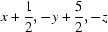
. *Cg*2 is the centroid of the C3–C8 benzene ring.

**Fig. 1.**
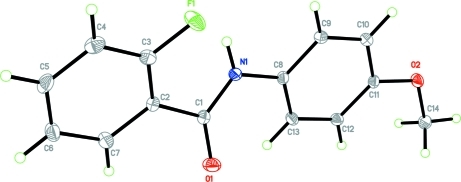
The structure of (I) with displacement ellipsoids for the non-hydrogen atoms drawn at the 50% probability level.

**Fig. 2.**
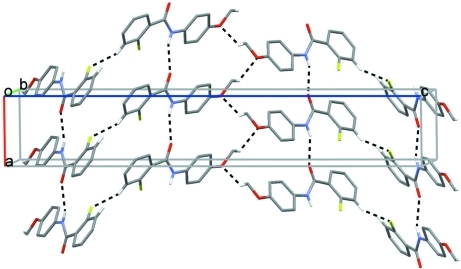
Crystal packing for (I) viewed down the b axis with hydrogen bonds drawn as dashed lines and H atoms not involved in hydrogen bonding omitted.

## supplementary crystallographic information

### Comment

The benzanilide core is present in compounds with a wide range of biological activity and benzanilides and benzamides are also used extensively in organic synthesis (Saeed *et al.*, 2008). Various N-substituted benzamides exhibit potent antiemetic activity (Vega-Noverola *et al.*, 1989). A one-pot conversion of 2-nitro-n-arylbenzamides to 2,3-dihydro-1*H*-quinazoline-4-ones has also been reported (Yoo *et al.*, 2005).

As part of our work on the structure of benzanilides and related compounds, we report here the structure of the title 2-fluorobenzamide derivative, I, Fig 1. In the structure of (I), the C2···C7 and C8···C13 rings are inclined at 27.06 (7)° and 23.86 (7)° respectively to the C2/C1/O1/N1/C8 amide portion of the molecule and at 3.46 (9)° to one another. The methoxy substituent lies close to the methoxybenzene ring plane with a maximum deviation of 0.152 (3) Å for C14. *N*-aryl 2-fluorobenzamide derivatives are reasonably common and bond distances and angles in the present molecule agree well with those in similar structures (see for example Saeed *et al.* 2008, Chopra & Guru Row, 2008, Donnelly *et al.* 2008, Cockroft *et al.* (2007), Spitaleri *et al.* 2004).

In the crystal structure intermolecular N1—H1N···O1 hydrogen bonds link molecules into rows along *a*. Weak C—H···O and C—H···F interactions further stabilize the packing, forming corrugated sheets in the *bc* plane (Table 1 and Fig.2).

### Experimental

2-Fluorobenzoyl chloride (5.4 mmol) in CHCl_3_ was treated with 4-methoxyaniline (21.6 mmol) under a nitrogen atmosphere at reflux for 3 h. Upon cooling, the reaction mixture was diluted with CHCl_3_ and washed consecutively with aq 1 *M* HCl and saturated aq NaHCO_3_. The organic layer was dried over anhydrous magnesium sulfate and concentrated under reduced pressure. Crystallization of the residue from CHCl_3_ afforded the title compound (81%) as white needles: Anal. calcd. for C_14_H_12_FNO_2_: C 68.56, H 4.93, N 5.71%; found: C 68.49, H 4.97, N 5.63%

### Refinement

The H atom bound to N1 was located in a difference map and refined isotropically. All other H-atoms were positioned geometrically and refined using a riding model with d(C—H) = 0.95 Å, *U*_iso_=1.2*U*_eq_ (C) for aromatic and 0.98 Å, *U*_iso_ = 1.5*U*_eq_ (C) for CH_3_ hydrogen atoms. In the absence of significant anomalous scattering effects, Friedel pairs were merged and a Flack parameter is not reported.

### Crystal data

**Table:**

C_14_H_12_FNO_2_	*F*(000) = 512
*M**_r_* = 245.25	*D*_x_ = 1.458 Mg m^−^^3^
Orthorhombic, *P*2_1_2_1_2_1_	Mo *K*α radiation, λ = 0.71073 Å
Hall symbol: P 2ac 2ab	Cell parameters from 5036 reflections
*a* = 5.2901 (2) Å	θ = 3.1–31.4°
*b* = 6.6435 (3) Å	µ = 0.11 mm^−^^1^
*c* = 31.7823 (11) Å	*T* = 89 K
*V* = 1116.98 (8) Å^3^	Rectangular, colourless
*Z* = 4	0.30 × 0.23 × 0.04 mm

### Data collection

**Table:**

Bruker APEXII CCD area-detector diffractometer	2185 independent reflections
Radiation source: fine-focus sealed tube	1980 reflections with *I* > 2σ(*I*)
graphite	*R*_int_ = 0.049
ω scans	θ_max_ = 31.5°, θ_min_ = 1.3°
Absorption correction: multi-scan (*SADABS*; Bruker, 2006)	*h* = −7→7
*T*_min_ = 0.853, *T*_max_ = 0.996	*k* = −8→9
19727 measured reflections	*l* = −46→45

### Refinement

**Table:**

Refinement on *F*^2^	Primary atom site location: structure-invariant direct methods
Least-squares matrix: full	Secondary atom site location: difference Fourier map
*R*[*F*^2^ > 2σ(*F*^2^)] = 0.035	Hydrogen site location: inferred from neighbouring sites
*wR*(*F*^2^) = 0.096	H atoms treated by a mixture of independent and constrained refinement
*S* = 1.06	*w* = 1/[σ^2^(*F*_o_^2^) + (0.0587*P*)^2^ + 0.1314*P*] where *P* = (*F*_o_^2^ + 2*F*_c_^2^)/3
2185 reflections	(Δ/σ)_max_ < 0.001
168 parameters	Δρ_max_ = 0.37 e Å^−^^3^
0 restraints	Δρ_min_ = −0.24 e Å^−^^3^

### Special details

**Table:**

Geometry. All e.s.d.'s (except the e.s.d. in the dihedral angle between two l.s. planes) are estimated using the full covariance matrix. The cell e.s.d.'s are taken into account individually in the estimation of e.s.d.'s in distances, angles and torsion angles; correlations between e.s.d.'s in cell parameters are only used when they are defined by crystal symmetry. An approximate (isotropic) treatment of cell e.s.d.'s is used for estimating e.s.d.'s involving l.s. planes.
Refinement. Refinement of *F*^2^ against ALL reflections. The weighted *R*-factor *wR* and goodness of fit *S* are based on *F*^2^, conventional *R*-factors *R* are based on *F*, with *F* set to zero for negative *F*^2^. The threshold expression of *F*^2^ > σ(*F*^2^) is used only for calculating *R*-factors(gt) *etc*. and is not relevant to the choice of reflections for refinement. *R*-factors based on *F*^2^ are statistically about twice as large as those based on *F*, and *R*- factors based on ALL data will be even larger.

### Fractional atomic coordinates and isotropic or equivalent isotropic displacement parameters (Å^2^)

**Table:**

	*x*	*y*	*z*	*U*_iso_*/*U*_eq_	
N1	0.8287 (2)	0.30490 (19)	0.12515 (4)	0.0120 (2)	
H1N	0.681 (4)	0.274 (3)	0.1327 (6)	0.018 (5)*	
C1	1.0253 (3)	0.1910 (2)	0.13830 (4)	0.0122 (3)	
O1	1.2460 (2)	0.21863 (18)	0.12733 (3)	0.0195 (2)	
C2	0.9628 (3)	0.0195 (2)	0.16750 (4)	0.0113 (2)	
C3	0.7617 (3)	0.0129 (2)	0.19568 (4)	0.0138 (3)	
F1	0.60670 (19)	0.17461 (15)	0.19944 (3)	0.0211 (2)	
C4	0.7144 (3)	−0.1504 (3)	0.22153 (4)	0.0172 (3)	
H4	0.5746	−0.1494	0.2403	0.021*	
C5	0.8740 (3)	−0.3153 (2)	0.21964 (5)	0.0175 (3)	
H5	0.8427	−0.4295	0.2369	0.021*	
C6	1.0809 (3)	−0.3137 (2)	0.19238 (5)	0.0176 (3)	
H6	1.1921	−0.4259	0.1912	0.021*	
C7	1.1234 (3)	−0.1472 (2)	0.16695 (4)	0.0151 (3)	
H7	1.2656	−0.1467	0.1487	0.018*	
C8	0.8450 (3)	0.4821 (2)	0.10044 (4)	0.0109 (2)	
C9	0.6527 (3)	0.6247 (2)	0.10514 (4)	0.0124 (3)	
H9	0.5154	0.5977	0.1236	0.015*	
C10	0.6600 (3)	0.8048 (2)	0.08330 (4)	0.0127 (3)	
H10	0.5290	0.9010	0.0869	0.015*	
C11	0.8609 (3)	0.8447 (2)	0.05591 (4)	0.0113 (3)	
C12	1.0490 (3)	0.7011 (2)	0.05019 (4)	0.0120 (3)	
H12	1.1830	0.7261	0.0310	0.014*	
C13	1.0418 (3)	0.5203 (2)	0.07255 (4)	0.0123 (3)	
H13	1.1716	0.4232	0.0687	0.015*	
O2	0.8549 (2)	1.02823 (15)	0.03604 (3)	0.0144 (2)	
C14	1.0690 (3)	1.0759 (2)	0.01050 (5)	0.0159 (3)	
H14A	1.2233	1.0662	0.0274	0.024*	
H14B	1.0517	1.2132	−0.0004	0.024*	
H14C	1.0788	0.9811	−0.0131	0.024*	

### Atomic displacement parameters (Å^2^)

**Table:**

	*U*^11^	*U*^22^	*U*^33^	*U*^12^	*U*^13^	*U*^23^
N1	0.0097 (5)	0.0108 (5)	0.0155 (5)	0.0003 (5)	0.0019 (4)	0.0030 (4)
C1	0.0124 (6)	0.0103 (6)	0.0138 (6)	−0.0004 (5)	0.0005 (5)	0.0013 (5)
O1	0.0117 (5)	0.0212 (6)	0.0255 (5)	0.0005 (4)	0.0013 (4)	0.0099 (5)
C2	0.0125 (6)	0.0102 (6)	0.0112 (5)	−0.0008 (5)	−0.0003 (5)	0.0000 (5)
C3	0.0124 (6)	0.0157 (6)	0.0132 (6)	0.0011 (5)	−0.0009 (5)	−0.0002 (5)
F1	0.0214 (5)	0.0231 (5)	0.0189 (4)	0.0094 (4)	0.0071 (4)	0.0027 (4)
C4	0.0154 (6)	0.0228 (8)	0.0134 (6)	−0.0033 (6)	0.0003 (5)	0.0036 (6)
C5	0.0194 (7)	0.0161 (7)	0.0169 (6)	−0.0054 (6)	−0.0028 (5)	0.0056 (6)
C6	0.0198 (7)	0.0132 (7)	0.0200 (6)	0.0019 (6)	−0.0024 (6)	0.0027 (6)
C7	0.0159 (6)	0.0139 (6)	0.0156 (6)	0.0020 (6)	0.0005 (5)	0.0006 (5)
C8	0.0117 (6)	0.0087 (5)	0.0122 (5)	0.0001 (5)	−0.0013 (5)	0.0005 (5)
C9	0.0108 (6)	0.0125 (6)	0.0140 (6)	0.0000 (5)	0.0022 (5)	−0.0004 (5)
C10	0.0116 (6)	0.0114 (6)	0.0150 (6)	0.0013 (5)	0.0006 (5)	−0.0002 (5)
C11	0.0124 (6)	0.0091 (6)	0.0123 (5)	−0.0005 (5)	−0.0018 (5)	0.0008 (5)
C12	0.0110 (5)	0.0123 (6)	0.0127 (5)	0.0003 (5)	0.0010 (5)	0.0009 (5)
C13	0.0119 (6)	0.0112 (6)	0.0139 (6)	0.0020 (5)	0.0013 (5)	0.0001 (5)
O2	0.0145 (5)	0.0108 (5)	0.0178 (5)	0.0019 (4)	0.0029 (4)	0.0042 (4)
C14	0.0146 (6)	0.0133 (6)	0.0199 (6)	−0.0009 (6)	0.0029 (6)	0.0047 (5)

### Geometric parameters (Å, °)

**Table:**

N1—C1	1.3522 (19)	C8—C13	1.3909 (18)
N1—C8	1.4177 (17)	C8—C9	1.3980 (19)
N1—H1N	0.84 (2)	C9—C10	1.3835 (19)
C1—O1	1.2317 (17)	C9—H9	0.9500
C1—C2	1.5066 (19)	C10—C11	1.3992 (19)
C2—C3	1.3914 (19)	C10—H10	0.9500
C2—C7	1.396 (2)	C11—O2	1.3734 (16)
C3—F1	1.3567 (17)	C11—C12	1.3902 (19)
C3—C4	1.384 (2)	C12—C13	1.3959 (19)
C4—C5	1.384 (2)	C12—H12	0.9500
C4—H4	0.9500	C13—H13	0.9500
C5—C6	1.396 (2)	O2—C14	1.4289 (18)
C5—H5	0.9500	C14—H14A	0.9800
C6—C7	1.388 (2)	C14—H14B	0.9800
C6—H6	0.9500	C14—H14C	0.9800
C7—H7	0.9500		
			
C1—N1—C8	126.09 (12)	C13—C8—N1	123.38 (12)
C1—N1—H1N	119.3 (14)	C9—C8—N1	117.36 (12)
C8—N1—H1N	114.6 (14)	C10—C9—C8	120.82 (13)
O1—C1—N1	123.93 (13)	C10—C9—H9	119.6
O1—C1—C2	119.66 (13)	C8—C9—H9	119.6
N1—C1—C2	116.40 (12)	C9—C10—C11	119.81 (13)
C3—C2—C7	116.65 (13)	C9—C10—H10	120.1
C3—C2—C1	126.02 (13)	C11—C10—H10	120.1
C7—C2—C1	117.31 (12)	O2—C11—C12	124.45 (12)
F1—C3—C4	117.34 (13)	O2—C11—C10	115.90 (12)
F1—C3—C2	119.61 (13)	C12—C11—C10	119.66 (12)
C4—C3—C2	123.02 (14)	C11—C12—C13	120.29 (13)
C3—C4—C5	118.98 (14)	C11—C12—H12	119.9
C3—C4—H4	120.5	C13—C12—H12	119.9
C5—C4—H4	120.5	C8—C13—C12	120.13 (13)
C4—C5—C6	119.95 (14)	C8—C13—H13	119.9
C4—C5—H5	120.0	C12—C13—H13	119.9
C6—C5—H5	120.0	C11—O2—C14	116.09 (11)
C7—C6—C5	119.60 (14)	O2—C14—H14A	109.5
C7—C6—H6	120.2	O2—C14—H14B	109.5
C5—C6—H6	120.2	H14A—C14—H14B	109.5
C6—C7—C2	121.77 (14)	O2—C14—H14C	109.5
C6—C7—H7	119.1	H14A—C14—H14C	109.5
C2—C7—H7	119.1	H14B—C14—H14C	109.5
C13—C8—C9	119.26 (13)		

### Hydrogen-bond geometry (Å, °)

**Table:**

*D*—H···*A*	*D*—H	H···*A*	*D*···*A*	*D*—H···*A*
N1—H1N···O1^i^	0.84 (2)	2.34 (2)	3.1366 (17)	158.5 (17)
C4—H4···F1^ii^	0.95	2.44	3.2472 (17)	143
C14—H14B···O2^iii^	0.98	2.61	3.3753 (18)	135

Symmetry codes: (i) *x*−1, *y*, *z*; (ii) −*x*+1, *y*−1/2, −*z*+1/2; (iii) *x*+1/2, −*y*+5/2, −*z*.
